# The Potential Role of Trained Immunity in Autoimmune and Autoinflammatory Disorders

**DOI:** 10.3389/fimmu.2018.00298

**Published:** 2018-02-20

**Authors:** Rob J. W. Arts, Leo A. B. Joosten, Mihai G. Netea

**Affiliations:** ^1^Department of Internal Medicine, Radboud Center for Infectious Diseases, Radboud University Medical Center, Nijmegen, Netherlands; ^2^Department of Medical Genetics, Iuliu Haţieganu University of Medicine and Pharmacy, Cluj-Napoca, Romania; ^3^Department for Genomics and Immunoregulation, Life and Medical Sciences Institute (LIMES), University of Bonn, Bonn, Germany

**Keywords:** epigenetics, monocytes, immunometabolism, innate immune memory, rheumatoid arthritis, systemic lupus erythematosus, Wegener’s granulomatosis, hyper Ig-D syndroom

## Abstract

During induction of trained immunity, monocytes and macrophages undergo a functional and transcriptional reprogramming toward increased activation. Important rewiring of cellular metabolism of the myeloid cells takes place during induction of trained immunity, including a shift toward glycolysis induced through the mTOR pathway, as well as glutaminolysis and cholesterol synthesis. Subsequently, this leads to modulation of the function of epigenetic enzymes, resulting in important changes in chromatin architecture that enables increased gene transcription. However, in addition to the beneficial effects of trained immunity as a host defense mechanism, we hypothesize that trained immunity also plays a deleterious role in the induction and/or maintenance of autoimmune and autoinflammatory diseases if inappropriately activated.

## Introduction

Since the introduction of the term trained immunity for the non-specific memory of the innate immune system in 2011 (1), an increasing number of studies have investigated its role in homeostasis and disease. Innate immune memory has been described in plants and non-vertebrates for a relatively long time, representing the immune adaptation in these species that lack an adaptive immune system (2). In vertebrates, it was firstly shown that NK cells possess a non-specific memory that contributes in the innate host defense (3, 4). Later also monocytes and macrophages were shown to have a non-specific memory (5, 6). When human monocytes were exposed *in vitro* with microbial components such as β-glucan of the *Candida albicans* cell wall or the Bacillus Calmette-Guérin (BCG) vaccine, and a week later restimulated with non-related stimuli, the capacity of cells to produce cytokines was increased compared with non-trained (naive) cells (3, 5). Moreover, when mice were challenged (or “trained”) with β-glucan or BCG *in vivo*, they showed lower mortality after lethal *C. albicans* or *Staphyococcus aureus* infections, a process that was largely dependent on the innate immune system (3, 5, 6). Moreover, in humans, BCG vaccination results in trained monocytes with increased responsiveness against microorganisms, which probably explains at least partly the lower mortality from a variety of infections in vaccinated children (3, 6–9).

There are a set of important characteristics that distinguish innate and adaptive immune memory processes. In case of the classical adaptive immune memory, a specific antigen is recognized and specific T- and/or B-lymphocytes expand that specifically respond to that antigen. The breadth of needed responses is insured by gene recombination of V-D-J system. Upon reinfection, long-term memory cells will respond very specifically to the same antigen: thus, adaptive memory is both specific and enhanced, compared to the primary response. In contrast, the molecular substrate of trained immunity is epigenetically mediated, with genome-wide changes in histone marks and thus chromatin architecture playing a major role in the change of the phenotype of monocytes and macrophages (6, 10). By stimulation of an innate immune cell (or its precursors) an enhanced non-specific immunological reaction will be evoked due to differences in which gene transcription takes place due to changes in chromatin configuration (11). Innate immune memory (trained immunity) will thus evoke an increased response, but which is non-specific.

Also changes in cellular metabolism of trained monocytes and macrophages were shown to be a major important component of the trained immunity phenotype. Induction of glycolysis in an mTOR/HIF-1α-dependent manner is indispensible for the induction of trained immunity (12, 13), just as induction of glutamine metabolism that results in fumarate accumulation (14, 15). Interestingly, there is a tight link between metabolic and epigenetic changes that occur in cells (16). We have for example recently shown, that induction of glycolysis is essential for the induction of epigenetic changes seen in trained immunity. When glycolysis was inhibited also the induction of trained immunity by its epigenetic changes was inhibited (12, 13). How induction of glycolysis exactly leads to epigenetic changes is still unclear, but accumulation of acetyl-CoA has been suggested as a mechanism, inducing histone acetylation (11). Moreover, also accumulation of fumarate (one of the intermediates of the TCA cycle) was shown to induce trained immunity by inhibiting histone demethylases and therefore inducing epigenetic changes in human monocytes (14). Recently, we have shown that induction of the cholesterol synthesis pathway, which results in mevalonate accumulation, is also one of the contributors to the induction of epigenetic changes in trained immunity (17). However, this remains a rather new topic of research and more research has to be performed to better understand the exact link between metabolism en epigenetics and its role in trained immunity.

Induction of trained immunity may be important for diseases characterized by defective function of innate immune responses. We have recently shown that the induction of trained immunity by β-glucan is able to counteract the epigenetic changes induced in monocytes in postsepsis immunoparalysis (18): this may represent a potential new therapy. However, trained immunity could also be the cause or play a role in maintaining disease activity in diseases characterized by excessive inflammation, although this needs to be investigated in future studies. In this review, we give an overview of literature that provides indications for the potential role of trained immunity in autoimmune and autoinflammatory diseases. We focus on the possibility that increased function of the myeloid cells in these conditions may be mediated by epigenetic rewiring, and thus possibly a trained immunity phenotype. Another possible mechanism how monocytes of patients with autoimmune and autoinflammatory diseases are more responsive is by specific genetic variations: this hypothesis has been extensively discussed in other reviews, and it will therefore be not presented here. Importantly, adaptive memory responses are also very well known to play a crucial part in autoimmune diseases. However, the role of the adaptive immune system in these diseases has been discussed elsewhere in very good recent review articles (19–21) and was therefore not included in this review.

## Rheumatoid Arthritis

Rheumatoid arthritis (RA) is one of the most common inflammatory arthritis. The pathogenesis of this autoimmune disease is complex and remains partially elusive; it is partly genetically regulated, but environmental factors also play a role, finally leading to synovial inflammation and destruction of bone and cartilage (22). Initially, the role of the adaptive immune system was mainly studied, as the loss of self tolerance of CD4^+^ T lymphocytes was considered the main defect in RA (23). However, this paradigm is changing and nowadays the focus has also shifted to the role of the innate immune system in RA (24–26). Innate immune cells have been defined as being the cause of the tissue-damaging inflammatory lesions, with macrophages as main producers of proinflammatory cytokines. Macrophages are considered the cause of cartilage and bone destruction by inducing inflammation and regulating osteoclast activity, e.g., by inducing RANKL production (27–30). The number of infiltrated macrophages seems to negatively correlate with response to therapy and the degree of joint erosion (31, 32) and patients with highly active RA show a greater amount of M1-type macrophages in the synovial fluid compared to those with lower disease scores (33). Targeting the cytokines produced by macrophages [e.g., tumor necrosis factor α (TNFα)] has been proven to be a very successful treatment (34).

When circulating monocytes from RA patients were analyzed, gene expression of several proinflammatory cytokines was increased (35, 36). CD14^+^ monocytes showed an increased expression of CD11b, and when *ex vivo* stimulated, increased production of IL-1β and IL-6 is observed (37). Interestingly, PI3K/mTOR and MAPK signaling pathways are also activated in RA monocytes (36, 38), and inhibiting mTOR reduced synovial osteoclast formation and protected against local bone erosions and cartilage loss (39, 40). In addition, epigenetic changes have been suggested to play a role, although no genome wide epigenetic analyses in monocytes/macrophages have been performed. In RA synovial tissue the HAT/HDAC balance is moved into the direction of histone acetylation (41). HDAC1 and HDAC2 activity has been specifically analyzed in synovial fibroblasts (not in synovial macrophages), but no difference was found (42). Interestingly, etanercept and adalimumab downregulate trimetylation of H3K4, H3K27, H3K36, and H3K79, as well as acetylation of histone 3 and 4 at the promoter site of CCL2 (MCP-1) in monocytes which correlated with RA disease activity (43). In contrast, a recent study did not identify an increase in H3K4me3 at *TNFA* and *IL6* genes of circulating monocytes in RA patients (44). In addition, the metabolic changes that occur in trained immunity also occur in macrophages from RA patients: upon stimulation with LPS higher levels of ATP are present in the patient-derived macrophages (45). Glycolysis is also upregulated, as are rate-limiting enzymes (e.g., PKM2, PFKFB3, and HK2) and the glucose transporters GLUT1 and GLUT3 (46). Functionally, glucose uptake and oxygen consumption are increased (46), whereas accumulation of glutamate, succinate, and fumarate are present as signs of a highly metabolic active state of an RA proinflammatory macrophage (47, 48), similar to a trained immunity phenotype (12, 14).

Hence, multiple similarities exist between tissue macrophages or circulating monocytes in RA and trained monocytes or macrophages. Interestingly, in RA patients an increased risk for atherosclerotic disease is seen (49), which corresponds with the hypothesis that trained macrophages contribute to the development of atherosclerotic lesions (50). How the training in RA is induced remains unknown, although several danger-associated molecular patterns (DAMPs), that could be released by local sterile inflammation and tissue damage in the joint have been proposed to induce trained immunity (51), just as the increased production of IL-32 (52). Future studies to investigate these mechanisms are warranted.

## Systemic Lupus Erythematosus (SLE)

In SLE several immunological abnormalities are present. The production of a number of antinuclear antibodies is the most prominent and well-known process, and they are used for diagnostic purposes. However, monocytes and macrophages also play a prominent role in the disease activity, which increasingly becomes a topic of research (53–55). In SLE patients, macrophages are characterized by a proinflammatory status and show an increased production of proinflammatory cytokines, such as IFNα, TNFα, and IL-6 (56–58). CD16^+^ monocytes express more CD80, CD86, HLA-DR, and CX3CR1 (59) and also expression data of CD14^+^ and non-classical monocytes demonstrate a proinflammatory phenotype (60–64). Inflammasome and interferon-regulated genes that were regulated by IRF1 (65), with IFNα as one of the main mediators (66–70), are induced in SLE monocytes. Moreover, monocytes show an improved antigen presentation capacity and are easily activated (71). It is therefore suggested that monocytes and macrophages are better capable of presenting self-antigens to autoreactive T cells and therefore inducing or maintaining disease activity (56). Interestingly, a SNP in the *IL1B* gene that was associated with lower IL-1β production upon LPS stimulation was protective for SLE (72).

Epigenetic modulation in monocytes clearly plays a role in SLE. Histones around the *TNFA* genomic region are highly acetylated in SLE monoyctes resembling high accessibility for transcription (73). Also whole-genome assessment of histone H4 of monocytes reveals hyperacetylation (74–76) and also at enhancers of monocytes SLE-specific alterations for H3K4me3 and H3K27me3 could be determined (77). Whole-genome epigenetic analysis of H3K4me3 of primary monocytes of SLE patients reveals a strong association with inflammation and immune responses related genes (78, 79). When H3K4me3 modulations were related to gene promoter site, and these were compared with the major epigenetically modulated promoter sites in *in vitro* β-glucan-trained monocytes (10), the M2 and M3 clusters (defined in β-glucan training) were also significantly modulated in SLE monocytes (Table 1). In another article in which monocytes from six SLE patients were compared with six controls, 136,573 DNase hypersensitivity sites (DHS) were defined. Of these DHS, 4,583 showed SLE-specific changes for H3K4me3 and 1,714 for H3K27me3 at promoter sites. At enhancer site theses numbers were 12,109 and 3,046, respectively (55). Transcription factor binding motifs analysis revealed PU.1 and CEBPB as main transcription factors related to the H3K4me3 induced genomic areas, just as seen for β-glucan-induced training (10). Also BLIMP1 and interferon-related transcription factors as STAT1/6, and IRF1/4/8 were defined (55).

**Table 1:** Comparison of H3K4me3-related GO-terms in β-trained monocytes and monocytes of SLE patients.

	Go-term	β-Glucan-induced trained immunity	SLE
		*p*-Value	*p*-Value
M1	Sugar binding	3.9E−2	0.72
M2	Carboxylic acid metabolic process	7.9E−5	4.5E−2
	Cellular ketone metabolic process	1.3E−4	0.32
	Oxidoreductase	1.4E−3	4.3E−2
	Lipid metabolic process	5.4E−6	3.2E−3
M3	Signal transducer activity	2.4E−3	3.7E−2
	Receptor activity	2.1E−2	1.2E−2
M4	Cofactor binding	3.5E−3	0.16
M5	Immune response	3.00E−19	3.7E−2
	Response to wounding	5.00E−17	1.3E−2
	Chemotaxis	5.2E−7	0.79
	Cytokine activity	8.00E−12	5.3E−2
	Chemokine activity	3.7E−9	9.3E−2

*H3K4me3 modulations were related to gene promoter site. Related GO-terms of the major epigenetically modulated promoter sites in *in vitro* β-glucan-trained monocytes, defined as M clusters (10), were compared with the same GO-terms in monocytes of SLE patients (78) and adjusted *p*-values are shown*.

In trained immunity induced by β-glucan and also by BCG, it has been shown that important changes in cellular metabolism are induced, and that changing concentrations of metabolites play a role in the modulation of epigenetic rewiring (10, 12–14).

In immune cells from SLE patients, metabolic changes are present and might influence the epigenetic landscape as well (80). SAMs are cofactors in DNA and histone methyltranferase reactions and in T cells in SLE SAMs have been shown to be modulated and influence the epigenetic landscape, both at DNA (81, 82) and histone methylation level (83, 84). Histone demethylases need α-ketogluterate and Fe^2+^ as cofactors, which were shown to be modulated in T cells of SLE patients (84). Acetyl CoA functions as an acetyl donor for histone acetyl transferases. Histone acetylation is defective in SLE, and when in mice in an SLE model HDACs were inhibited, this showed a positive effect on disease activity and nephritis (85–87).

Finally, also activation of the mTOR pathway is a marker of disease and of onset of disease in SLE. The mTOR pathway is activated in many immune cells, among others also in monocytes (88–90). Inhibition of mTOR with rapamycin has shown beneficial effects on several outcome measures, but monocytes/macrophages were unfortunately not part of the analysis [reviewed in Ref. (88)].

The modulation of monocytes from the peripheral blood could be due to changes induced in the bone marrow. Gene expression analysis of mononuclear bone marrow cells from SLE patients reveals that ERK, JNK, and p38 MAP kinases and STAT3 are significantly upregulated (91). As anti-dsDNA antibodies can bind to TLR4 and activate the NLRP3 inflammasome, it is tempting to speculate whether they might be the factor inducing trained immunity in SLE patients (92, 93).

## Sjögren’s Syndrome (SS)

Sjögren’s syndrome is a chronic autoimmune disease characterized by salivary and lacrimal gland dysfunction (94). Also in the case of SS, clues can be found in literature to suggest that a trained immunity profile may characterize its myeloid cells. Immune cell infiltrates of the salivary glands in SS mainly contain macrophages and DCs with increased IL-12 and IL-18 expression and depletion of macrophages in the gland tissue improved disease symptoms in a mouse model (95–97). When peripheral CD14^+^ monocytes were stimulated with apoptotic cells, they showed an increased production of TNFα or IL-1β and a decreased production of IL-10, thus displaying a proinflammatory profile (98, 99). Also when monocyte-derived dendritic cells were stimulated with LPS a clear proinflammatory profile was seen, with among others higher levels of TNFα, MIG, IFNα, IL-6, IL-12, MIP1α/β, MCP1 compared to healthy volunteers (100). In non-stimulated circulating monocytes, type I IFN-related genes were higher expressed compared to control monocytes, which correlated with the induction of B-cell activating factor (BAFF) (101–103). When monocytes were stimulated with IFNγ, increased IL-6 and BAFF production was seen as well (104). However, others have reported different effects, with decreased cytokine production upon PPD stimulation of PBMCs isolated from SS patients (105). Furthermore, NFκB activation is promoted by reduced IκBα expression in Sjögren monocytes (106). Increased STAT1 is seen in SS monocytes (107), similar to constitutive STAT5 activation (108).

No epigenetic assessment of monocytes from SS patients has been performed, apart from increased expression of miRNA 146a and 181a in PBMCs (109–113), and miR-34b-3p, miR-4701-5p, miR-609, miR-300, miR-3162-3p, and miR-877-3p upregulation in monocytes. These miRNAs collectively suppress TGFβ signaling, as opposed to proinflammatory interleukin-12 and Toll-like receptor/NFκB pathways (114). Analysis of cellular metabolic processes and induction of mTOR activation in monocytes/macrophages in SS has not been performed, but treatment with a rapamycin nanoparticle reduced destruction of lacrimal glands in a mouse model (115).

## Behçet’s Disease (BD)

Behçet’s disease is an inflammatory disorder that is characterized by oral en genital lesions, arthritis, and uveitis. The exact cause of BD is unknown (116). Myeloid cells play an important role in Behçet’s disease activity, as granulocyte and monocyte adsorption apheresis reduced disease symptoms in two patients (117). Furthermore, peripheral monocytes of BD patients are activated and produce more proinflammatory cytokines, which might be a first clue for the presence of a trained immunity phenotype in monocytes of BD patients (118–122). The P2X7 receptor, involved in the activation of IL-1β production, has an increased expression on monocytes in BD, which is regulated by TNFα (123), while the expression of TLR2 and TLR4 is also upregulated (124, 125). The response of CD14^+^ monocytes from BD patients to IFNγ showed increased production of CXCL9 and CXCL10; however, the CXCL10 production might be increased due to dysregulated posttranslational regulation (126). A whole-genome DNA methylation profiling of monocytes revealed 383 CpG sites to be differently regulated compared to healthy controls (and only 123 were found in CD4^+^ T cells), with cytoskeleton modulation as one of the main regulated pathways (127).

## Systemic Sclerosis (SSc)

Systemic sclerosis is a complex autoimmune disease with extensive fibrosis, vascular alterations and immune activation among its principal features (128). Also in SSc the monocyte compartment appears to play an important role. CD14^+^ monocytes levels are increased in peripheral blood and in skin infiltrates (129, 130). An increased type I IFN signature was observed in early and definite SSc patient monocytes, which correlated with increased BAFF mRNA expression. Production of other cytokines, chemokines and their receptors (among others IL6, TNFα, and TGFβ) is upregulated as well (131–145). Monocytes of SSc patients produce more reactive oxygen species (146, 147), whereas nitric oxide production was decreased (148). SSc PBMCs produce more IL-8 and CCL18 when stimulated with IgG (149). In the vascular alterations present in SSc patients, monocytes appear to play a role as they can differentiate into fibroblast-like cells and produce extracellular matrix (modulating) proteins, resulting in a proangiogenic but impaired vasculogenic environment (132, 150–156). The macrophage activation markers CD163 and CD204 are more expressed in SSc (129, 132), which by some authors has been linked to a M2 macrophage phenotype with anti-inflammatory and profibrotic features (157). The alveolar macrophages in SSc patients with pulmonary fibrosis have a strong M2 phenotype with expression of CCL17, CCL18, and CCL22 and increased activation of STAT 3 (158). Increased TNFα production by these cells was also reported (159).

On the level of epigenetic profiles, hardly any data are available in monocytes or macrophages from SSc patients. The only data available in monocytes show that histone demethylation plays a role in the production of tissue inhibitor of metalloproteinases 1 in the presence of TLR8 stimulation (160). However, given the broad amount of data present in the literature, an epigenetic analysis on circulating monocytes in SSc would be a logical next step.

## Wegener’s Granulomatosis (WG)

Wegener’s granulomatosis is a systemic inflammatory disorder characterized by vasculitis of the small- and medium-size vessels in many organs. The exact cause remains unknown, but monocytes have been suggested to play a role in disease activity (161).

Monocytes of WG patients are activated, as shown by increased CD11b and CD64 expression, and increased concentrations of neopterin and IL-6 are found in the serum (162). The expression of adhesion molecules is increased on monocytes from WG patients (163). Interestingly, anti-PR3 induces cytokine production by monocytes and it was shown that when monocytes are primed with ANCA or PR3 antibodies, their responses to LPS and LTA stimulation increase, with more TNFα, IL-6 and IL-8 production (164) and increased expression of CD14, CD18, and several PRRs (165, 166). Monocytes of WG patients that did not respond to methotrexate showed increased intracellular levels of IL-12 and TNFα (but no IL-8) that normalized after cyclophosphamide treatment (167). Also IL-8, IL-12, and MCP-1 production was increased in WG monocytes (166, 168–170). In contrast, other studies have shown that monocytes from WG patients are shown to produce less reactive oxygen species and have impaired phagocytosis (171) and in one report, monocytes of WG patients were stimulated with LPS, they produced less TNFα compared to healthy controls (172). No studies on epigenetic modulation of cellular metabolism of monocytes in WG are available. In order to assess whether a trained immunity phenotype is indeed responsible for the functional changes observed, such studies should be performed.

## Sarcoidosis

Sarcoidosis is a complex systemic granulomatous disorder of unknown etiology. Circulating monocytes op sarcoidosis patients produce less IL-10 (173), express more CD16^+^ (174, 175), BAFF (166), TLR2 and TLR4 (176), IL-2R (177), adhesion molecules (178), produce more proinflammatory cytokines (176, 179–181) and oxygen radicals (182), and have increased phagocytic activity (183), thus showing an increased activated phenotype (184). Moreover, monocytes of sarcoidosis patients are more likely to form giant cells (185). Fibrin, which is newly formed in granulomas, is able to induce IL-1β production and might therefore serve as an inductor of trained immunity (186).

RNA-sequencing analysis of monocytes of sarcoidosis patients revealed several differentially expressed genes, with enrichment of ribosome, phagocytosis, lysosome, proteasome, oxidative phosphorylation, and metabolic pathways are the main pathways (187). No epigenetic analysis of monocytes in sarcoidosis has been done so far (188).

## Type 1 Diabetes Mellitus (T1DM)

Type 1 diabetes mellitus is an important autoimmune disease resulting in profound defects in glucose metabolism. The exact underlying mechanism is unknown, but autoimmune destruction of β-cells of the pancreas is the cause of insulin deficiency (189). Monocytes and macrophages are thought to play a key role in the development of T1DM (190, 191). When peripheral blood monocytes from recent-onset T1DM patients were assessed, more CD14^+^CD16^+^ monocytes were found, which was negatively correlated with insulin and C-peptide serum levels. Furthermore, these monocytes showed higher expression of HLA-DR and CD86, and showed an activated proinflammatory phenotype (192–196). In recent-onset T1DM patients, plasma TNFα levels were higher which correlated with CD14 expression (197). However, another study showed decreased total number of monocytes in T1DM (198). Serum levels of MIF and MCP-1 are also increased (199). Prior to development of T1DM, children show an IFN transcriptional signature in PBMCs, which might be a result of a recent upper respiratory infection. Increased expression of SIGLEC-1 on CD14^+^ monocytes was also found (200). Furthermore, it was shown that IL-1β plays a central role in the inflammation seen in T1DM (201) and that during the development of T1DM TLR-induced IL-1β and IL-6 production from monocytes is enhanced (202). Importantly, inhibiting IL-1β production or IL-1β signaling can improve T1DM outcome (203–206). In contrast, monocyte-derived DCs did not appear to be affected in recent-onset T1DM (191).

Epigenetics has been proposed to play a role in T1DM as well (207). Assessment of DNA methylation profiles of 32 T1DM patients versus 31 healthy individuals revealed 153 hypomethylated and 225 hypermethylated loci in whole blood, and, respectively, 155 and 247 in monocytes. However, whether these are causative to the disease or a consequence of the pathological process remains unclear (208). In another study methylation variable positions were assessed in monocytes prior to development of T1DM: 132 positions were identified and associated with a gene, with important immunology-related genes as HLA-DQB1, HLA-DRB1, NFKB1A, and TNF as major examples (209). Also important histone modifications were seen in H3K9ac in monocytes, but also these were more likely to be induced after development of the disease (210). However, in one study, H3K9ac at HLA-DRB1 and HLA-DQB1 of monocytes was shown to correlate with T1DM susceptibility prior to disease (211). Differences in H3K9me3 in inflammatory and autoimmune-related pathways were found in lymphocytes of T1DM patients, but no differences were found in monocytes (212).

Cellular metabolism of monocytes in T1DM prior to disease onset is still poorly known. Only one study revealed changes in the transcriptional signature of cell metabolism, cell survival, and oxidative stress in monocytes of recent-onset DM1. Interestingly, one of the main induced genes was HIF1A (213), although mTOR does not appear among the significantly differently regulated genes.

Lastly, as BCG is able to induce trained immunity, it could be expected that BCG-vaccinated children are at higher risk to develop T1DM. However, in an observational trial no such correlation has been found (214, 215). Interestingly, in a randomized controlled trial, BCG was suggested to have a beneficial effect on insulin production, as induction of TNF production resulted in reduced autoimmune phenotype of innate immune cells and induction of Tregs (216, 217).

## Autoinflammatory Disorders

Autoinflammatory disorders or periodic fever syndromes consist of a set of diseases that are characterized by periodic episodes of fever and inflammation. Common symptoms are joint pain, rash, abdominal pain, and long-term disease can result in amyloidosis. These diseases are not induced by autoantibodies or autoreactive T-cells but are the result of a hyperfunctional innate immune system, which is due to genetic defects. The familial autoinflammatory syndromes are generally rare, and the pathogenesis is not well understood for some of them (218). Here, we argue that trained immunity could be a likely contributor in these diseases.

Tumor necrosis factor receptor-associated periodic syndrome (TRAPS) is a multisystemic autoinflammatory condition associated with heterozygous TNFRSF1A mutations, presenting with a variety of clinical symptoms, many of which still unexplained. TRAPS monocytes shown an inflammatory baseline state, with enhanced IL1B and IL1R1 gene expression, also in non-active disease, whereas IFN and TGFβ are downregulated (219, 220). Also CD16 expression is upregulated (221) and MAPK can be spontaneously activated (220). Interestingly, a TRAPS patient with a monocytic fasciitis has been successfully treated with tacrolimus (an mTOR inhibitor) (222).

Cryopyrin-associated periodic syndromes (CAPS) are caused by a mutation in NLRP3 inflammasome, resulting in increased IL-1β and IL-18 production, but impaired production of the anti-inflammatory IL-6 and IL-1RA cytokines (223, 224). Interestingly, although the genetic defect is the main cause of these abnormalities, epigenetic analysis of CAPS monocytes revealed that DNA methylation was also affected, resulting at increased expression of inflammasome-related genes. When CAPS patients were treated with IL-1 neutralizing therapies, their methylation profile reversed toward that of healthy controls (225).

In familial Mediterranean fever (FMF), *ex vivo* LPS-stimulated PBMCs and monocytes produce more IL-1α and β, IL-6, IL-8, IL-12, IL-18, and TNFα, and in non-stimulated PBMCs higher production of IL-6 and TNFα was found (226–230), and also expression of CD11b was increased (231).

Hyper-IgD syndrome (HIDS) is an autoinflammatory disorder caused by a mutation in mevalonate kinase (232, 233), in which patients experience periodic attacks of sterile inflammation with symptoms such as fever, skin lesions, lymphadenopathy, and arthralgia (234). Interestingly, PBMCs of HIDS patients produce more cytokines in unstimulated as well as stimulated state and the IgD itself was able to induce cytokine production (235–238). In a whole-blood transcriptome analysis several glycolysis-related genes were higher expressed in HIDS patients, which decreased after canakinumab treatment (239). In addition, we recently have shown broad genome-wide changes in the H3K27Ac marker in monocytes of HIDS patients, which are due to mevalonate accumulation and are believed to express a trained immunity profile (Bekkering et al., 2018 Cell in press).

The common factor in these autoinflammatory syndromes is overproduction of IL-1β, which is one of the reasons why anti-IL-1 therapeutic approaches are successful (240). As IL-1β is considered as a causal factor and plausibly plays a role in maintaining disease, accumulating evidence suggests that IL-1β is an important inducer of trained immunity. Interestingly, old studies have shown that injection of IL-1β in mice prevented death from subsequent bacterial and fungal infections (241, 242). It is tempting to hypothesize that IL-1β induces trained immunity and prevent from the subsequent infections, by epigenetically modifying monocytes and macrophages resulting in more proinflammatory immune cells. To further substantiate this hypothesis, we have recently performed experiments in which human monocytes are *ex vivo* trained with IL-1β. This indeed resulted in trained monocytes that produced more IL-6 and TNFα upon restimulation with LPS, and also increased H3K4me3 occupancy at the promoters of *IL6* and *TNFA* was observed (Arts et al., Cell Host Microbe 2018, in press). While IL-1 can induce beneficial effects in infections through induction of trained immunity, a negative consequence could be induction of overinflammation, which might result in an autoinflammatory disease. This thus opens a potential new field of research, where trained immunity in these autoinflammatory diseases should be further evaluated.

## Chronic Granulomatous Disease (CGD)

Chronic granulomatous disease is an inherited immunological disorder, in which intracellular superoxide radical production is deficient. Although CGD is an immunodeficiency, it also has autoinflammatory characteristics, which is why it is discussed here as well. Normally CGD presents in the first years of life with severe recurrent bacterial and fungal infections, but it can also present later in life (243). CGD phagocytes are impaired in destroying phagocytozed microorganisms, rendering the patients susceptible to bacterial and fungal infections. Besides this immunodeficiency, CGD patients suffer from various auto inflammatory symptoms, such as granuloma formation and Crohn-like colitis (244). Also monocytes from CGD patients display a proinflammatory phenotype with increased secretion of inflammasome-mediated cytokines (IL-1β, IL-18) possibly due the inflammasome triggering effect of ROS, but also increases of other cytokines and chemokines, and NKκB and ERK expression upon stimulation (245–247). Circulating monocytes display an inflammatory phenotype with more CD16^+^ expression and more intracellular IL-1β and TNFα (247). However, others have shown lower TNFα production by CGD monocytes (248). Incubation of CGD monocytes with rapamycin (an mTOR inhibitor) counterbalanced the preactivation state of monocytes *ex vivo* (247), hence implicating a role for mTOR. IL-1 inhibition reduced inflammation in humans and reduced disease activity of, e.g., CGD-associated colitis, possibly also by restoring autophagy (249, 250). Interestingly, injection of fungal β-glucan results in hyperinflammation and necrosis in CGD mice associated with increased IL-1β, IL-6, and TNFα production (251–253).

Metabolically also clear differences were found in CGD monocytes. Several metabolites of the tryptophan pathway accumulate and indoleamine 2,3-dioxygenase is activated (254), just as seen in monocytes stimulated with LPS or IFNγ (255). CGD monocytes have been shown to higher acidification (256), which might be the result of increased lactate production.

## Conclusion

In this review, we present an overview of the data supporting the concept that monocytes from patients with several autoimmune and autoinflammatory diseases display features consistent with a trained immunity phenotype. The phenotype of a trained monocyte has been defined with characteristics as (1) increased cytokine production, (2) changes in cellular metabolism (mainly increased glycolysis and lactate production), and (3) epigenetic rewiring (Table 2). Trained immunity could serve a role in the initiation of the disease and in the maintenance or aggravation of the symptoms. In the case of disease initiation, a genetic or environmental factor (or combinations of both) would induce trained monocytes/macrophages that initiate the disease. In the case of disease progression, monocytes/macrophages become trained and are therefore easier activated, which would result in the maintenance or deterioration of disease symptoms. This is an important distinction to take into account, as different experimental approaches would apply.

**Table 2:** Overview of trained immunity-related patterns in autoimmune and autoinflammatory diseases.

	Cytokines and chemokines	Metabolism of immune cells	Epigenetic marks	mTOR signaling	Others
Rheumatoid arthritis	Circulating monocytes have increased expression of proinflammatory cytokines (35, 36)*Ex vivo*-stimulated monocytes produce more IL-1β and IL-6 (37)	Higher ATP levels upon LPS stimulation of macrophages (45)Upregulated glycolysis (46)Increased oxygen consumption (46)Accumulation of succinate in macrophages (47)Accumulation of succinate, fumarate, glutamate in synovial fluid (48)	H3K4me3 at *TNFA* and *IL6* is not induced in monocytes (44)	PI3K/mTOR signaling pathway and MAPK are activated in RA monocytes (36, 38)	Increased CD11b expression on CD14 circulating monocytes (37)

SLE	Cirulating monocytes produce more proinflammatory cytokines (56–58)SNP in the *IL1B* gene was protective for SLE (72).	No studies specific on monocytes	Histones around *TNFA* are highly acetylated in monoyctes (73)histone H4 of monocytes is hyperacetylated (74–76)H3K4me3 of SLE monocytes are associated with inflammation and immune response-related genes (78, 79)SLE-specific H3K4me3 and H3K27me3 (enhancer) modifications (55, 77)	Activated mTOR pathway in monocytes/macorphages (88–90)Rapamycin inhibition improves outcome (88)	CD16^+^ monocytes express more CD80, CD86, HLA-DR and CX3CR1 (59)CD14^+^ and non-classical monocytes display a proinflammatory phenotype (60–64)Inflammasome and interferon-regulated genes are induced in moncytes (65–70)monocytes show an improved antigen presentation capacity (71)ERK, JNK, and p38 MAP kinases and STAT3 are significantly upregulated in mononuclear bone marrow cells (91).

Sjögren	CD14^+^ monocytes stimulated with apoptotic cells show increased TNFα and IL-1β, and decreased IL-10 production (98, 99).Monocyte-derived DCs produce more proinflammatory cytokines (100)Higher expression of IFN I-related genes, which correlated with BAFF (101–103)IFNγ-stimulated monocytes produce more IL-6 and BAFF (104)	?	Several miRNAs are upregulated in monocytes (114)	?	NFκB activation is promoted by reduced IκBα expression in monocytes (106)Increased STAT1 activation (107)Constitutive STAT5 activation (108)

Behçet’s disease	Peripheral monocytes are activated and produce more proinflammatory cytokines (118–122)Increased production of CXCL9 and 10 upon IFNγ stimulation (126).	?	DNA methylation profiling of monocytes revealed 383 CpG sites to be differently regulated in monocytes (127)	?	Increased P2X7 receptor (123), and TLR2 and 4 expression (124, 125)

Systemic	Monocytes display an increased IFN type I signature, but also other cytokines, chemokines and their receptors are upregulated (131–145)A SNP in TLR2, which results in increased production of TNFα and IL-6 of monocytes, was associated with SSc (257)	?	?	?	Monocytes produce more ROS (146, 147), whereas NO production is decreased (148)Increased expression of CD163 and CD204 (129, 132)

Wegener’s granulomatosis	Increased IL-6 expression (162)Increased production of several proinflammatory cytokines (164, 166, 168–170)	?	?	?	Increased CD11b and CD64 expression (162) and CD14, CD18, and several PRRs (165, 166)Increased expression of adhesion molecules (163)

Sarcoidosis	Higher production of proinflammatory cytokines (176, 179–181)	RNA-sequencing of monocytes shows enrichment of oxidative phosphorylation and metabolic pathways (187)	?	?	Higher expression of CD14^+^CD16^+^ (174, 175), BAFF (166), TLR2 and 4 (176), IL-2R (177), adhesion molecules (178)Higher production of oxygen radicals (182) and increased phagocytic activity (183)More likely to form giant cells (185)

T1DM	Higher plasma levels of TNF, MCP-1, and MIF (197, 199), and TLR-induced IL-1β and IL-6 production by monocytes is increased (200–202). IL-1β inhibition improves T1DM outcome (203–206)	Gene expression of recent-onset T1DM monocytes shows signature with cellular metabolism and oxidative stress as main pathways, and with HIF1A among the induced genes (213)	Several DNA hypo and hypermethylated loci were defined in T1DM monocytes (208, 209). H3K9ac marks are correlated with T1DM (210, 211)	?	More CD14 + CD16 + monocytes in recent-onset T1DM patients, with higher HLA-DR and CD86 expression and proinflammatory phenotype (192–196).

TRAPS	Enhanced IL1B and IL1R1, and decreased IFN and TGFB expression (219, 220)	?	?	Monocytic fasciitis successfully treated with tacrolimus (222)	Upregulated CD16 expression (221)Spontaneous MAKP activation (220)

CAPS	Increased IL-1β and IL-18 production, Production of Il-6 and IL-1RA appears to be impaired (223, 224)	DNA methylation was affected, resulting at increased expression of inflammasome-related genes (225)	?	?	?

FMF	LPS-stimulated PBMCs and monocytes produce more IL-1α and β and non-stimulated PBMCs produce more of IL-6 and TNFα (226–228)	?	?	?	Higher expression of CD11b (231)

HIDS	PBMCs produce more cytokines in unstimulated or stimulated state (235–238)	?	?	?	?

CGD	Monocytes display a proinflammatory phenotype with increased secretion of IL-1β and IL-18, but also other cytokines and chemokines (245–247)More IL-1β and TNFα expression (247)	Metabolites of the tryptophan pathway accumulate and indoleamine 2,3-dioxygenase (IDO) is activated (254)Monocytes show higher acidification (256)	?	Incubation of monocytes with rapamycin counterbalanced the preactivation state (247)	Increased NK-κB and ERK expression upon stimulation (246, 247)More CD16^+^ expression (247)

By providing a molecular mechanism in the described diseases in terms of trained immunity, we inherently describe potential novel therapies. For certain components of the metabolic pathways and epigenetic pathways described to be important for trained immunity, specific and non-specific inhibitors are already available and new ones are being developed. We have shown before that by inhibiting specific metabolic pathways or by specifically inhibiting certain epigenetic modulating enzymes, the induction of trained immunity can be counteracted (12–15). Hence, by identifying the specific trained immunity pathways that play a role in the induction and progression of disease activity in these autoinflammatory and autoimmune diseases, it is hoped that novel targeted immunotherapies will be developed.

However, all data presented here are circumstantial and do not prove a causal relation between the disease symptoms and monocyte function. Therefore, specifically applied experiments on the role of trained immunity in these diseases are essential to further unravel the role of trained immunity. By elucidating the potential role of trained immunity in these (but supposedly also other) diseases, new steps can be made in better understanding the pathophysiology of these diseases. Even more importantly, this could potentially lead to new approaches for therapeutic intervention in these diseases.

## Author Contributions

RA wrote the first draft and LJ and MN made revisions.

## Conflict of Interest Statement

The authors declare that the research was conducted in the absence of any commercial or financial relationships that could be construed as a potential conflict of interest.
